# Forecasting magma-chamber rupture at Santorini volcano, Greece

**DOI:** 10.1038/srep15785

**Published:** 2015-10-28

**Authors:** John Browning, Kyriaki Drymoni, Agust Gudmundsson

**Affiliations:** 1 Department of Earth Sciences, Royal Holloway University of London, Egham, TW20 0EX, United Kingdom; 2 Department of Mineralogy and Petrology, National and Kapodistrian University of Athens, Greece

## Abstract

How much magma needs to be added to a shallow magma chamber to cause rupture, dyke injection, and a potential eruption? Models that yield reliable answers to this question are needed in order to facilitate eruption forecasting. Development of a long-lived shallow magma chamber requires periodic influx of magmas from a parental body at depth. This redistribution process does not necessarily cause an eruption but produces a net volume change that can be measured geodetically by inversion techniques. Using continuum-mechanics and fracture-mechanics principles, we calculate the amount of magma contained at shallow depth beneath Santorini volcano, Greece. We demonstrate through structural analysis of dykes exposed within the Santorini caldera, previously published data on the volume of recent eruptions, and geodetic measurements of the 2011–2012 unrest period, that the measured 0.02% increase in volume of Santorini’s shallow magma chamber was associated with magmatic excess pressure increase of around 1.1 MPa. This excess pressure was high enough to bring the chamber roof close to rupture and dyke injection. For volcanoes with known typical extrusion and intrusion (dyke) volumes, the new methodology presented here makes it possible to forecast the conditions for magma-chamber failure and dyke injection at any geodetically well-monitored volcano.

Santorini is a volcanic island built predominantly by lava effusion and dome-forming eruptions^1^, periodically interrupted by catastrophic ignimbrite-forming eruptions^2^. The most recent caldera-forming event occurred approximately 3650 years ago (at 3.6 ka) and is commonly referred to as the Minoan eruption. Since that eruption Santorini has experienced primarily effusive activity, located centrally in the caldera complex, which over time has formed the Kameni islands^1^. Nea Kameni (Fig. 1) produced at least three well documented eruptive episodes during the 20^th^ century^1^. The volume of magma extruded during each individual event is estimated from the subaerial shapes and sizes of the lava flows and domes^1^. Volumes of older submarine eruptions have also be estimated using bathymetric data^1^,^3^. The Kameni islands lie along the Kameni line^4^, a tectonic lineament which may influence magma emplacement and caldera faulting^5^. The average volume of magma issued during each individual effusive eruption is around 0.06 km^3^. This is much smaller than the estimated volume of magma involved in Santorini’s caldera forming events at 3.6 ka and ~26 ka with the dense-rock equivalent (DRE) volumes of 20–30 km^3^ 2. Whilst spectacular and impressive, the 20^th^ century eruptions posed little risk to the majority of Santorini’s inhabitants. However, the islands are now a major tourist destination with a summer population in excess of 50,000^6^. Even a small future eruptive event coupled with caldera-wall instabilities could therefore have negative consequences.

In January 2011 Santorini volcano entered a period of unrest, meaning that the ground surface began inflating^3^,^7^,^8^,^9^ and the magnitude and frequency of earthquakes increased^3^,^4^,^5^,^7^,^8^ . This period lasted until April 2012 when signs of unrest ceased. The unrest was triggered by magma being transported as a dyke (a fluid-driven fracture) from great depths (>10 km) below the surface to a much shallower (~4 km deep) magma chamber^3^,^7^,^8^,^9^. Using geodetic techniques, it is estimated that a combined volume of approximately 0.021 km^3^ (21 million cubic metres) of magma entered the shallow magma chamber, presumably in two main phases, in just over one year^3^. None of the geodetic or seismic signals indicate that magma rose from the shallow chamber as a dyke towards the surface, suggesting that increased pressure in the shallow chamber due to the volume of new magma was insufficient to rupture the chamber roof. But how close to rupture was the chamber? To answer that question for Santorini and other well-monitored volcanoes, we provide a model to calculate the excess pressure in the chamber following the receipt of new parental magma.

## Results

In the simplest terms, a magma chamber roof will rupture when^10^,^11^,^12^


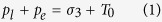


where *p*_*l*_ is the lithostatic or overburden pressure (due to the weight of the overlying rocks), *p*_*e*_ is the magmatic excess pressure within the chamber, *σ*_*3*_ is the local minimum compressive principal stress and *T*_*0*_ is the local tensile strength of the host rock. Since *σ*_*3*_ is the local stress, at the margin of the chamber, stress-concentration effects due to magma-chamber shape and loading are automatically taken into account in Eq. (1)^11^,^12^. Typical values of solid-rock tensile strengths range from 0.5 to 6 MPa, the most common values being 3–4 MPa^11^,^13^. It follows from Eq. (1) that for a part of the chamber to fail in tension the local value of *p*_*e*_ must during an unrest period reach *T*_*o*_. At any other time the chamber is considered to be in lithostatic equilibrium with the surrounding host rock, in which case the excess pressure *p*_*e*_ is zero (this assumption is discussed in the section Methods). Evidence for the mechanism of chamber rupture comes from fracture mechanics principles and field observations of extinct and now fossil magma chambers, in Iceland and elsewhere, some of which have the well-exposed roofs dissected by dykes^12^.

### Common intrusive (dyke) volumes at Santorini volcano

**Figure 1:**
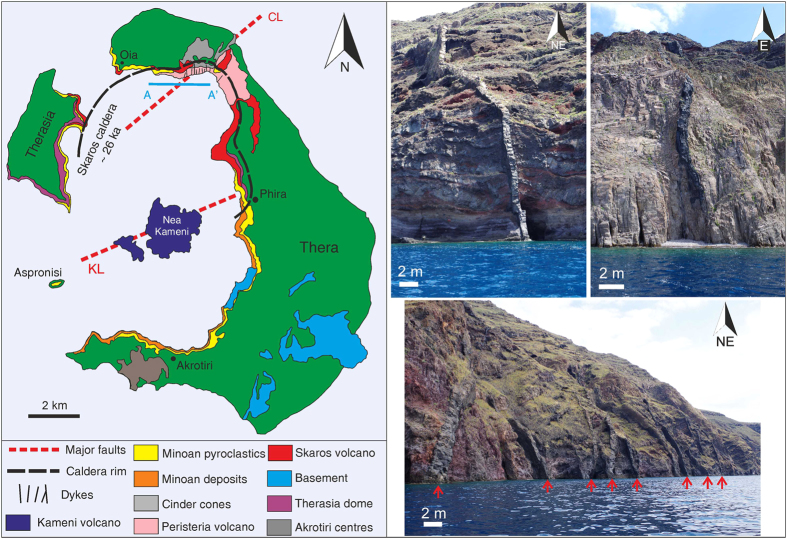
Simplified geological map of Santorini. Showing two main tectonic elements: the Kameni and Coloumbo lines, the inferred Skaros caldera rim, and the approximate location of dykes within the northern caldera wall. All the exposed dykes are located along the northernmost extent of the Skaros caldera wall and the island of Therasia; some are marked in the photographs with red arrows. Most dyke measurements were taken from a boat along the profile A–A′. The stratigraphy of the caldera is complex, being made up of many different types and ages of deposits. Many dykes within the wall are arrested, i.e. are non-feeders. Santorini geological map is modified from^29^. Photos: John Browning.

**Figure 2:**
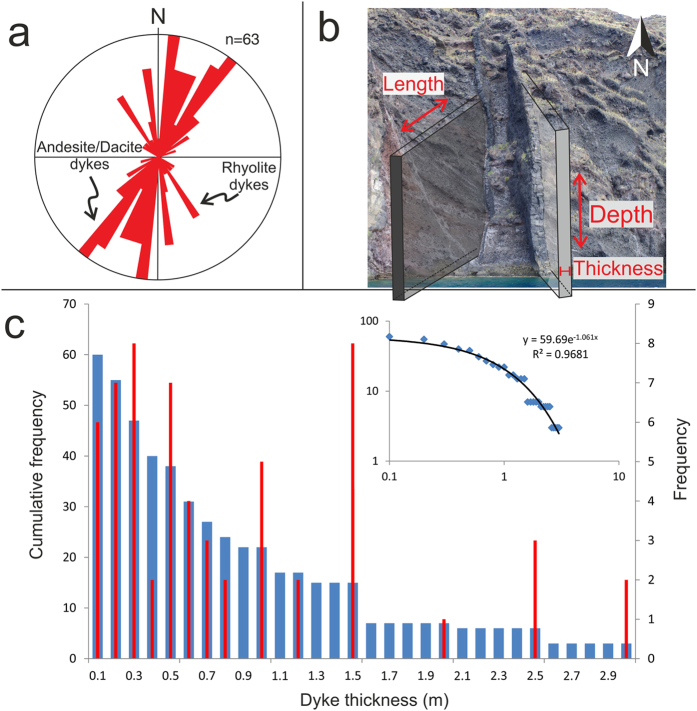
(**a**) Orientation and (**c**) thickness of 63 dykes in (**b**) the northern caldera wall of Santorini. Most dykes are less than 1.5 m thick and strike dominantly NE-SW; those dykes which strike NW-SE generally tend to be thicker and composed of felsic magmas. The average thickness of dykes measured is 1 m, the minimum being 0.1 m and the maximum 5 m. For visualisation purposes the thickest dyke shown is 3 m. (**c**) Dykes thicknesses plotted as cumulative frequency distributions follow an exponential trend (blue bars). Individual dyke measurements plotted as a histogram with bin size 0.1 m are shown as red bars^28^. Photo: John Browning.

Geological exposures at Santorini also offer insights into the dynamics of magma movement within the volcano over time. At least 63 dykes (frozen or solidified magma-filled fractures) can be observed cutting the scalloped caldera wall in the northernmost part of the island of Thera (Fig. 1). The dykes range from andesite to trachydacite in composition^14^ and are primarily exposed over a narrow section of the caldera wall at around 3.5 km east of the town of Oia and south of Finikia. The caldera wall is accessible by boat, and abseiling in some parts, making it possible to measure the thickness (roughly the palaeo-aperture or dyke-fracture opening) and the strike and dip (attitude) of the dykes. Dykes strike dominantly NE-SW (Fig. 2), matching the inferred strike of the Coloumbo line, a tectonic lineament which connects the Santorini volcanic complex to the nearby Coloumbo volcano^15^,^16^. Fifteen dykes strike NW-SE. These dykes tend to be thicker and lighter in colour, indicating a more evolved (felsic) composition. The thickest of the NE-striking dykes is 2 m, the average thickness being 0.7 m. By contrast, the thickest of the NW-striking dykes is about 5 m, the average thickness being 1.7 m. Dyke thicknesses fit an exponential scaling law when plotted as a cumulative frequency distribution (Fig. 2). Alternatively, the finding may reflect the relatively small dataset or indicate two power-law sets with different scaling exponents—larger data sets normally suggest dyke thicknesses following power-laws^11^. The dykes are predominantly sub-vertical, dipping on average around 80°. The dip of individual dykes, however, varies considerably, indicating local stress variations in the host rock^17^.

In order to estimate the volume of magma contained within any one individual dyke generated from a shallow magma chamber at Santorini caldera we assume a dyke-length (along strike or strike dimension) to thickness ratio of 1500^10^,^11^. This ratio, a common value based on measurements of dykes worldwide, is used because it is not possible to measure the lateral extent of any dykes within the caldera at Santorini. The assumed ratio also takes into account that dykes tend to become longer at greater depths because of general increase in Young’s modulus (*E*) with crustal depth^11^. Many dyke tips are seen, suggesting that most of the dykes within the caldera wall are non-feeders, i.e. did not supply magma to an eruption but rather became arrested at contacts between dissimilar layers within the volcano. Arrested dykes, non-feeders, are commonly observed in well-exposed outcrops such as caldera walls and cliffs^18^,^19^, as in Santorini, indicating that magma-chamber rupture and dyke injection is no guarantee for an eruption. In particular, Santorini has a complex geologic stratigraphy made up of many rock units and layers with contrasting mechanical properties^1^,^17^ (Fig. 1) whose contacts tend to arrest dykes^11^,^17^.

There is little difference between the thicknesses of the feeder-dykes and non-feeders (arrested dykes)^18^. Since dyke thickness is linearly related to the dyke strike and dip dimensions^11^, we use an average dyke dimension when calculating the volume of magma transported out of the chamber during common eruptions. Using an average dyke thickness of 1 m, then, based on the length/thickness ratio above, the average length or strike-dimension is 1500 m. Similarly, based on the geodetically determined depth to the present magma chamber (about 4000 m), the average dyke depth or dip-dimension is 4000 m. Using these dimensions and a thickness of 1 m, the average dyke volume is 0.006 km^3^. This average dyke volume can then be combined with the known average volume of material erupted during the Santorini’s 20^th^ century eruptions to estimate the volume of the shallow source chamber and the necessary added volume needed to rupture the chamber and inject a new dyke.

### Estimating the volume of Santorini’s magma chamber

The total volume *V*_*m*_ of a shallow chamber located within host rock of average compressibility *β*_*r*_ and tensile strength *T*_*0*_ is related to the total volume *V*_*e*_ of magma leaving (being squeezed out of) the chamber to produce the eruptive materials and/or the injected dyke through the equation^10^,^12^,^20^


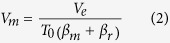


where *β*_*m*_is the magma compressibility. Using a typical shallow-crustal compressibility of 3 × 10^−11^ Pa^−1^ 12, an average *in-situ* tensile strength of 3.5 MPa^6^, and magma compressibility of 1.25 × 10^−10^ Pa^−1^ 10, then Eq. (2) reduces to


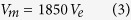


Here we use an average value taken from experimentally derived ranges for compressibility of various magmas and compressibility and tensile strength of host rocks^6^,^10^,^12^, assuming a totally molten magma chamber. Many magma chambers may be partly compartmentalised with zones of differential volatile concentrations and crystal mushes, in which case they should be modelled as poro-elastic. These and related topics are discussed further in the section Methods.

Using the estimated average volume of a typical individual dyke within the Santorini caldera, 0.006 km^3^, and the average measured volume of magma erupted for a typical individual eruptive phase on the Kameni islands, 0.06 km^3^, then *V*_*e*_ in Eq. (3) becomes 0.066 km^3^. It follows then from Eq. (3) that the total volume *V*_*m*_ of the shallow chamber active during these eruptions is about 122 km^3^. For a penny-shaped or sill-like chamber, as are common^12^, and based on the dimensions of the three caldera structures which make up Santorini, the chamber radius would be about 4 km and the thickness about 2 km. The geometry may, of course, be different. We do not aim to constrain the precise chamber geometry, since it is not needed for the present purpose. The main points are to assess the trade of between radius and thickness and to show that, for the estimated volume, the chamber must be so large as to encompass a significant area of the present-day caldera.

### Magma-chamber rupture during recharge

Since the excess pressure at the time of magma-chamber rupture is normally equal to the local tensile strength at the rupture site (Eq. 1), we can substitute *p*_*e*_ for *T*_*0*_ in Eq. (2). Also, assuming that the volume added to the chamber before rupture Δ*V*_*m*_ is roughly equal to the magma volume leaving the chamber following the rupture *V*_*e*_, we can rewrite Eq. (2) as


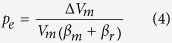


Here it is assumed that before the new magma of volume *∆V*_*m*_ entered the chamber (from a deeper source or reservoir), the chamber was in lithostatic equilibrium with the host rock and its excess pressure *p*_*e*_ thus zero. This is a normal assumption for periods of quiescence and follows partly because unrest (e.g., inflation and earthquakes) would be expected in case of rising *p*_*e*_ (*p*_*e*_ > 0) whereas quiescence periods are characterised by the absence of unrest signals^10^,^12^.

**Figure 3:**
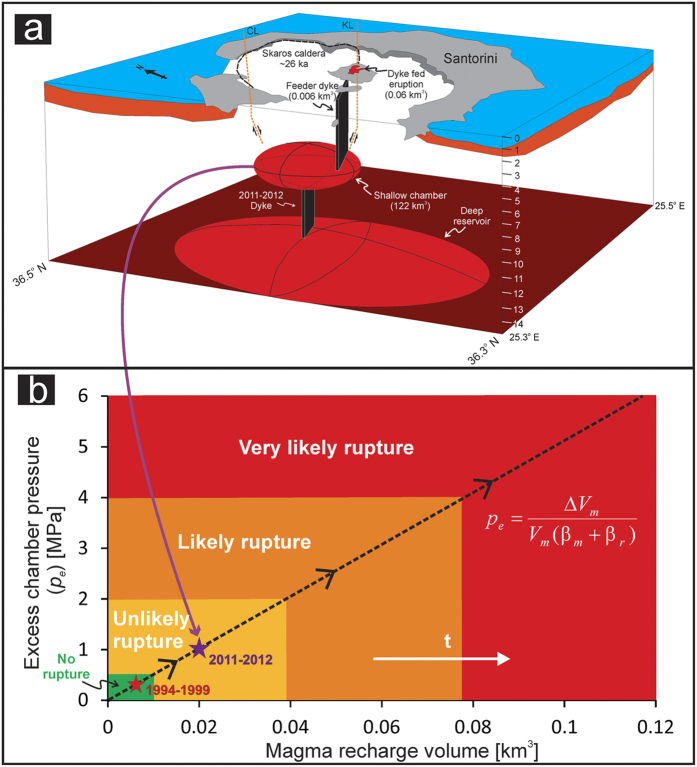
(**a**) Simplified 3D model of Santorini volcanic system based on geodetic^3^,^4^,^7^,^8^,^9^ and geological data. A deep reservoir feeds magma into a shallow system at around 4 km depth; this shallow chamber has a current total volume of approximately 122 km^3^. The volume is estimated using the average volume of dykes (0.006 km^3^) and the average volume of 20^th^ century eruptions (0.06 km^3^) together with fracture-mechanics and continuum-mechanics principles. The exact nature of the Kameni and Colombo tectonic lineaments is unclear, but here both are drawn as normal faults. The box is drawn between 25.3–25.5° E and 36.3–36.5° N to a depth of 15 km below the surface. (**b**) Excess pressure (*p*_*e*_) within the shallow magma chamber at Santorini as a function of the volume of new magma (Δ*V*_*m*_) entering the chamber from a deeper source over time. Here the results are applied to the shallow chamber of Santorini based on the estimated size of the chamber. The method, however, can be applied to any active central volcano for which (1) there exist extrusion (lava and pyroclastic flows) and intrusion (primarily dyke) volume estimates and (2) geodetic data as to inflation volumes. Rupture probability statements based on increasing excess pressure within the shallow chamber allow forecasts of dyke formation to be made in real time during magma recharge events. The model has been applied to the inflation episodes of 1994–1999 (red star) and 2011–2012 (purple star).

During the 2011–12 unrest period in Santorini, the volume of new magma that entered the shallow chamber Δ*V*_*m*_ is estimated at around 0.021 km^3^ [Ref. 7]. Substituting this in Eq. (4) and using the above values for the size of the chamber and the compressibilities, the corresponding excess pressure *p*_*e*_ in the chamber increased from zero to 1.1 MPa during the unrest period. Our results indicate that whilst the total amount of new magma which entered the shallow chamber during the 2011–2012 unrest period at Santorini represents a very small fraction (~0.02%) of the estimated total magma stored, the excess pressure increase within that shallow chamber came close to the surrounding host rock’s tensile strength^10^, and therefore close to rupturing the chamber boundary and injecting a dyke (Fig. 3). For completeness we also consider the slow inflation episode of 1994–1999 where the volume of new magma that entered a chamber to the north of the caldera was estimated at around 0.78 × 10^−5^ km3 [Ref. 21]. For the five year period we estimate the excess pressure increase within the shallow chamber as about 0.3 MPa. In all the unrest episodes, even if the chamber boundary ruptures and injects a dyke, the local stresses within the edifice ultimately govern whether the dyke becomes arrested or, alternatively, reaches the surface to supply magma to an eruption^17^ at Nea Kameni or elsewhere in Santorini.

Most models used to explain periods of unrest at Santorini simulate one shallow magma chamber pressure centre north of the Kameni islands^3^,^7^,^8^,^9^. Other models, however, relate the unrest to two shallow magma sources^4^,^21^,^22^, some citing the anomalous distribution of seismicity along the Kameni line and a separate pressure source at a depth of 1 km, or possibly 5.5 km further north in association with the 1994–1999 inflation. Our calculated volume constraints easily incorporate the area of the proposed two magma sources^4^,^21^. Two chambers are thus not needed in our model—a single, moderately large and partly compartmentalised^12^ chamber is sufficient—but our results certainly do not rule out that possibility. Focussed seismicity on the Kameni line during periods of unrest may be related to its mechanical properties being different from those of the surrounding crust, resulting in stress concentration along the line, or deep-seated reservoirs. Further considerations of that topic, however, are outside the scope of the present paper.

Eruptions at Santorini volcano are mostly with volumes ≪0.1 km^3^. However, much larger eruptions, with volumes >30 km^3^, occur occasionally and presumably from the same magma chamber. For a chamber with a volume of some 122 km^3^, a large fraction (about one-fourth) of its magma must be squeezed out to generate such a large eruption. Ordinary elastic and poro-elastic models of the type described here cannot explain such large magma removal from the chamber. The forced chamber volume reduction during piston-like caldera collapse, however, is apparently able to squeeze out a high proportion of the magma in the chamber, thereby explaining occasional large eruptions from moderately sized chambers. Then the large-volume eruptions are not the cause but rather the consequence of the caldera collapse^23^. Combining the ordinary poro-elastic mechanism with the collapse-driven mechanism, the estimated moderately large shallow chamber at Santorini volcano can supply magma to both small and large eruptions.

## Conclusion

The methodology presented here and applied to Santorini volcano can be used alongside real-time geodetic observations to help forecast magma chamber rupture at any geodetically well-monitored volcano. This new method, therefore, represents a valuable first-order tool for volcano observatories during periods of volcanic unrest. Further steps must be taken in order to better constrain the local stresses within the shallow parts of volcanic edifices, as these provide primary control on dyke propagation paths. As yet no comprehensive model exists to ascertain whether a dyke injected from a ruptured magma chamber will reach the surface and supply magma to an eruption. Even so, estimating the volume of magma stored at shallow depths and the conditions required to mobilise that magma are important steps in the development of reliable volcano-tectonic models for forecasting volcanic eruptions.

## Methods

Dyke measurements at Santorini were conducted during a five day field campaign in April 2014. Dykes dissecting the northern caldera wall were measured directly on land as well as from a boat. Outcrops of dykes are mostly limited to the northern caldera wall and parts of Therasia. Dyke attitudes (strike and dip) were measured using a compass clinometer and thicknesses and morphological data of some dykes were measured directly in the field, but mostly spotted from the boat at a distance of around 10–15 m.

Lava flow volumes are taken directly from previous studies^1^,^7^. Here we average all of the known lava flow volumes to obtain the individual eruption average of 0.06 km^3^. Maximum and minimum lava volumes are given in the Supplementary Data.

Excess pressure (*p*_*e*_) is derived from the difference between total fluid pressure (*p*_*t*_) within the chamber and the lithostatic stress (*p*_*l*_) where


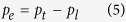


For lithostatic equilibrium, an assumed condition when the chamber is not undergoing unrest, all the principal stresses at the chamber boundary are equal (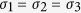) and equal to the lithostatic stress (*p*_*l*_). It then follows from Eq. (1) that 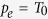, which is used to derive Eq. (4). The assumption of lithostatic equilibrium is valid because any pressure deviation from lithostatic results in stress concentration in the host rock of the chamber, and thus in volcanic unrest as would be reflected in e.g., geodetic changes and seismicity. Even relatively small-scale unrests are detected on well-monitored volcanoes, such as the creep-like inflation during 1994–1999 at Santorini^22^. When the chamber ruptures and injects a dyke the overpressure or driving pressure *p*_*o*_ in that dyke is given by^11^


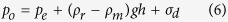


where *p*_*e*_ is the excess magma pressure in the magma chamber, *ρ*_*r*_ is the average host rock density, *ρ*_*m*_ is the average dyke-magma density, *g* is acceleration due to gravity (9.81 ms^−2^), *h* is the height of the dyke above its contact with the chamber (or the dip dimension of the dyke), and *σ*_*d*_ is differential stress, i.e. the difference between vertical stress and the minimum principal horizontal stress in the rock layer where the dyke overpressure is calculated. Note that this formulation includes the effects of gravity. The opening displacement of dykes and the depth to magma chamber intersection can also be calculated analytically^24^ but is not within the scope of the current work.

To calculate the ratio of erupted material to volume of the shallow magma chamber we assumed the chamber to be totally molten. This is the standard assumption used in the inversion of geodetic data to infer the depths to magma chambers associated with inflation and deflation (unrest) periods^25^. However, many chambers contain volatiles and crystals and therefore may be closer to a poro-elastic material, in which case Eq. (4) becomes modified to^10^,^12^,^26^


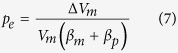


where *β*_*p*_ is the pore compressibility of the chamber, i.e. the fractional change in pore volume (magma fraction) of the chamber for unit change in the excess pressure. In this case, when new magma is received by the chamber (from the deeper source), the new magma is partly accommodated through compression of the old magma and partly by expanding chamber pore space. Compression of old magma leads to an increase in magmatic pressure (+*p*_*e*_), whereas pore expansion leads to a decrease in magmatic pressure (−*p*_*e*_). The excess pressure increase as new magma is added to the chamber then depends on the values of the pore and magma compressibilities. The magma compressibility, however, is generally much higher than either the host-rock compressibility (Eq. 4) or the pore compressibility (Eq. 7). It follows that the calculated excess pressure for a given addition of new magma to the chamber depends primarily on the magma compressibility, and the results are similar when using Eqs (7 and 4) for shallow magma chambers. Our model assumes that magma compressibility remains constant throughout an unrest period and is homogeneously distributed. More data is needed on magma compressibilities and their variations, and until such data become available the present assumption has to be made, as is the case in most deformation studies^3^,^7^. Also, we focus on the magma chamber compressibility as a whole. Therefore, whilst variations in compressibility almost certainly exist in compartmentalised chambers^12^, and will influence aspects of associated localised volume changes, when the chamber is treated as a single homogeneous system our assumptions are justified.

More specifically, there are no doubt significant uncertainties or errors in the estimated compressibilities of the rocks and the magmas used in eqs (2, 3, 4 and 7). The calculated compressibilities are based on earlier data provided by Murase and McBirney^20^. However, no uncertainties are provided for these original data, so that the standard propagation of uncertainties or errors estimates, whereby the uncertainties or errors add in quadrature^27^, cannot be made. In contrast to the compressibilites, which may vary considerably, the *in-situ* tensile strengths may be regarded as close to constant. The most common values are 3–4 MPa^11^, so that the average value of 3.5 MPa, used here, has an uncertainty or error of about 0.5 MPa, or less than 15%. A rough estimate of the total error in the excess pressure estimates, based on the assumptions used, would suggest an uncertainty of perhaps 50%.

Dyke thicknesses are plotted on a histogram (Fig. 2) with bin widths of 0.1 m together with cumulative frequency distributions where the probability *P(x)* that *x* has a value greater than or equal to *x*, is given by^28^,


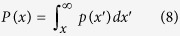


## Additional Information

**How to cite this article**: Browning, J. *et al.* Forecasting magma-chamber rupture at Santorini volcano, Greece. *Sci. Rep.* **5**, 15785; doi: 10.1038/srep15785 (2015).

## Supplementary Material

Supplementary Information
