# Supplementary material for: Forecasting magma-chamber rupture at Santorini volcano, Greece
Source: Sci Rep. 2015 Oct 28;5:15785. doi: 10.1038/srep15785 (PMC4623603; doi:10.1038/srep15785)
Supplement: Supplementary Information [file srep15785-s1.doc]

**Forecasting magma-chamber rupture at Santorini volcano, Greece**

**John Browning^1^*, Kyriaki Drymoni^1&2^  and Agust Gudmundsson^1^***

^1^Department of Earth Sciences, Royal Holloway University of London, Egham, TW20 0EX, United Kingdom

^2^Department of Mineralogy and Petrology, National and Kapodistrian University of Athens, Greece

*e-mail: [john.browning.2012@live.rhul.ac.uk](mailto:john.browning.2012@live.rhul.ac.uk); [rock.fractures@googlemail.com](mailto:rock.fractures@googlemail.com)

**Supplementary data:**

| Kameni island lava volumes^1,3^ | | |
| --- | --- | --- |
| **Eruption (name or date)** | **Duration (days)** | **Average volume (km^3^)** |
| Konus | - | 0.00065 |
| 1950 | 23 | 0.000009 |
| 1939 | 682 | 0.01054 |
| 1925 | 949 | 0.082 |
| NK | - | 0.057 |
| 1866 | 1723 | 0.17 |
| 1707 | 1575 | 0.11 |
| 1570 | - | 0.068 |
| Drakon | - | 0.0164 |
| NK East | - | 0.044 |
| 726 | - | 0.02 |
| 46 | - | 0.139 |
|  | **Total Average Volume (km^3^)** | **0.06** |
